# Lazy hazy days

**DOI:** 10.15252/embr.202255545

**Published:** 2022-06-27

**Authors:** Howy Jacobs

**Affiliations:** ^1^ Tampere University Tampere Finland; ^2^ La Trobe University Melbourne Australia

**Keywords:** Biotechnology & Synthetic Biology, Economics, Law & Politics, Evolution & Ecology

## Abstract

Scientists have warned about the looming climate crisis for decades, but the world has been slow to act. Are we in danger of making a similar mistake, by neglecting the dangers of other climactic catastrophes?

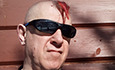

On one of my trips to Antarctica, I was enjoined to refer not to “global warming” or even to “climate change.” The former implies a uniform and rather benign process, while the second suggests just a transition from one state to another and seems to minimize all the attendant risks to survival. Neither of these terms adequately or accurately describes what is happening to our planet's climate system as a result of greenhouse gas emissions; not to mention the effects of urbanization, intensive agriculture, deforestation, and other consequences of human population growth. Instead, I was encouraged to use the term “climate disruption,” which embraces the multiplicity of events taking place, some of them still hard to model, that are altering the planetary ecosystem in dramatic ways.

With climate disruption now an urgent and undeniable reality, policymakers are finally waking up to the threats that scientists have been warning about for decades. They have accepted the need for action (UNFCCC Conference of the Parties, 2021), even if the commitment remains patchy or lukewarm. But to implement all the necessary changes is a massive undertaking, and it is debatable whether we have enough time left. The fault lies mostly with those who resisted change for so long, hoping the problem would just go away, or denying that it was happening at all. The crisis situation that we face today is because the changes needed simply cannot be executed overnight. It will take time for the infrastructure to be put in place, whether for renewable electricity, for the switch to carbon‐neutral fuels, for sustainable agriculture and construction, and for net carbon capture. If the problems worsen, requiring even more drastic action, at least we do have a direction of travel, though we would be starting off from an even more precarious situation.

However, given the time that it has taken—and will still take—to turn around the juggernaut of our industrial society, are we in danger of making the same mistakes all over again, by ignoring the risks of the very opposite process happening in our lifetime? The causes of historic climate cooling are still debated, and though we have fairly convincing evidence regarding specific, sudden events, there is no firm consensus on what is behind longer‐term and possibly cyclical changes in the climate.

The two best‐documented examples are the catastrophe of 536–540 AD and the effects of the Laki Haze of 1783–1784. The cause of the 536–540 event is still debated, but is widely believed to have been one or more massive volcanic eruptions that created a global atmospheric dust‐cloud, resulting in a temperature drop of up to 2°C with concomitant famines and societal crises (Toohey *et al*, 2016; Helama *et al*, 2018). The Laki Haze was caused by the massive outpouring of sulfurous fumes from the Laki eruption in Iceland. Its effects on the climate, though just as immediate, were less straightforward. The emissions, combined with other meteorological anomalies, produced a disruption of the jetstream, as well as other localized effects. In northwest Europe, the first half of the summer of 1783 was exceptionally hot, but the following winters were dramatically cold, and the mean temperature across much of the northern hemisphere is estimated to have dropped by around 1.3°C for 2–3 years (Thordarson & Self, 2003). In Iceland itself, as well as much of western and northern Europe, the effects were even more devastating, with widespread crop failures and deaths of both livestock and humans exacerbated by the toxicity of the volcanic gases (Schmidt *et al*, 2011).

Other volcanic events in recorded time have produced major climactic disturbances, such as the 1816 Tambora eruption in Indonesia, which resulted in “the year without a summer,” marked by temperature anomalies of up to 4°C (Fasullo *et al*, 2017), again precipitating worldwide famine. The 1883 Krakatoa eruption produced similar disruption, albeit of a lesser magnitude, though the effects are proposed to have been much longer lasting (Gleckler *et al*, 2006).

Much more scientifically challenging is the so‐called Little Ice Age in the Middle Ages, approximately from 1250 to 1700 AD, when global temperatures were significantly lower than in the preceding and following centuries. It was marked by particularly frigid and prolonged winters in the northern hemisphere. There is no strong consensus as to its cause(s) or even its exact dates; nor even that it can be considered a global‐scale event rather than a summation of several localized phenomena. A volcanic eruption in 1257 with similar effects to the one of 1816 has been suggested as an initiating event. Disruption of the oceanic circulation system resulting from prolonged anomalies in solar activity is another possible explanation (Lapointe & Bradley, 2021). Nevertheless, and despite an average global cooling of < 1°C, the effects on global agriculture, settlement, migration and trade, pandemics such as the Black Death and perhaps even wars and revolutions, were profound.

Once or twice in the past century, we have faced devastating wars, tsunamis and pandemics that seemed to come out of the blue and exacted massive tolls on humanity. From the most recent of each of these, there is a growing realization that, although these events are rare and poorly predictable, we can greatly limit the damage if we prepare properly. Devoting a small proportion of our resources over time, we can build the infrastructure and the mechanisms to cope, when these disasters do eventually strike.

Without abandoning any of the emergency measures to combat anthropogenic warming, I believe that the risk of climate cooling needs to be addressed in the same way. The infrastructure for burning fossil fuels needs to be mothballed, not destroyed. Carbon capture needs to be implemented in a way that is rapidly reversible, if this should ever be needed. Alternative transportation routes need to be planned and built in case existing ones become impassable due to ice or flooding. Properly insulated buildings are not just a way of saving energy. They are essential for survival in extreme cold, as those of us who live in the Arctic countries are well aware—but many other regions also experience severe winters, for which we should all prepare.

Biotechnology needs to be set to work to devise ways of mitigating the effects of sudden climactic events such as the Laki Haze or the Tambora and Krakatoa eruptions, as well as longer‐term phenomena like the Little Ice Age. Could bacteria be used, for example, to detoxify and dissipate a sulfuric aerosol such as the one generated by the Laki eruption? Methane is generally regarded as a major contributor to the greenhouse effect, but it is short‐lived in the atmosphere. So, could methanogens somehow be harnessed to bring about a temporary rise in global temperatures to offset short‐term cooling effects of a volcanic dust‐cloud?

We already have a global seed bank in Svalbard (Asdal & Guarino, 2018): It might easily be expanded to include a greater representation of cold‐resistant varieties of the world's crop plants that might one day be vital to human survival. And, the experience of the Laki Haze indicates a need for varieties capable of withstanding acid rains and other volcanic pollutants, as well as drought and water saturation. An equivalent (embryo) bank for strains of agriculturally important animals potentially threatened by the effects of abrupt cooling of the climate or catastrophic toxification of the atmosphere is also worth considering.

It has generally been thought impractical and pointless to prepare for even rarer events, such as cometary impacts, but events that have occurred repeatedly in recorded history and over an even longer time scale (Helama *et al*, 2021) are likely to happen again. We should and can be better prepared. This is not to say that we should pay attention to every conspiracy theorist or crank, or paid advocates for energy corporations that seek short‐term profits at the expense of long‐term survival, but the dangers of climate disruption of all kinds are too great to ignore. Instead of our current rather one‐dimensional thinking, we need an “all‐risks” approach to the subject: learning from the past and the present to prepare for the future.

## Disclosure and competing interests statement

The author declares that he has no conflict of interest.
